# A Correlation Analysis of Geomagnetic Field Characteristics in Geomagnetic Perceiving Navigation

**DOI:** 10.3389/fnbot.2021.785563

**Published:** 2021-12-23

**Authors:** Hong Li, Junsuo Qu, Xiangkui Jiang, Yun Niu

**Affiliations:** ^1^School of Automation, Xi'an University of Posts and Telecommunications, Xi'an, China; ^2^Xi'an Key Laboratory of Advanced Control and Intelligent Process, School of Automation, Xi'an University of Posts and Telecommunications, Xi'an, China; ^3^School of Marine Science and Technology, Northwestern Polytechnical University, Xi'an, China

**Keywords:** animal geomagnetic perception, geomagnetic navigation, correlation analysis, hexpath algorithm, word magnetic model, world magnetic model

## Abstract

It is well-known that geomagnetic fields have multiple components or parameters, and that these geomagnetic parameters are related to each other. In this paper, a parameter selection method is proposed, and this paper mainly discusses the correlation of geomagnetic field parameters for geomagnetic navigation technology. For the correlation analysis between geomagnetic parameters, the similarity calculation of the correlation coefficient is firstly introduced for geomagnetic navigation technology, and the grouped results are obtained by data analysis. At the same time, the search algorithm (Hex-path algorithm) is used to verify the correlation analysis results. The results show the same convergent state for the approximate correlation coefficient. In other words, the simulation results are in agreement with the similarity calculation results.

## Introduction

Lots of evidence has indicated that many kinds of animals can achieve long-distance and goal-oriented navigation without pinpoint accuracy (Walker et al., 2002). This is due to the existence of the 'geomagnetic sense' (Walker et al., 1997). Kramer states that animals could firstly determine their position relative to the goal and set the course for their goal by the Earth's geomagnetic field (Kramer, 1953). It is reported that pigeons and sea turtles can reach a destination by sensing geomagnetic information (Rodda, 1984; Dennis et al., 2007). These animals can locate homing and foraging areas depending on their perception of the geomagnetic field.

**Figure 1:**
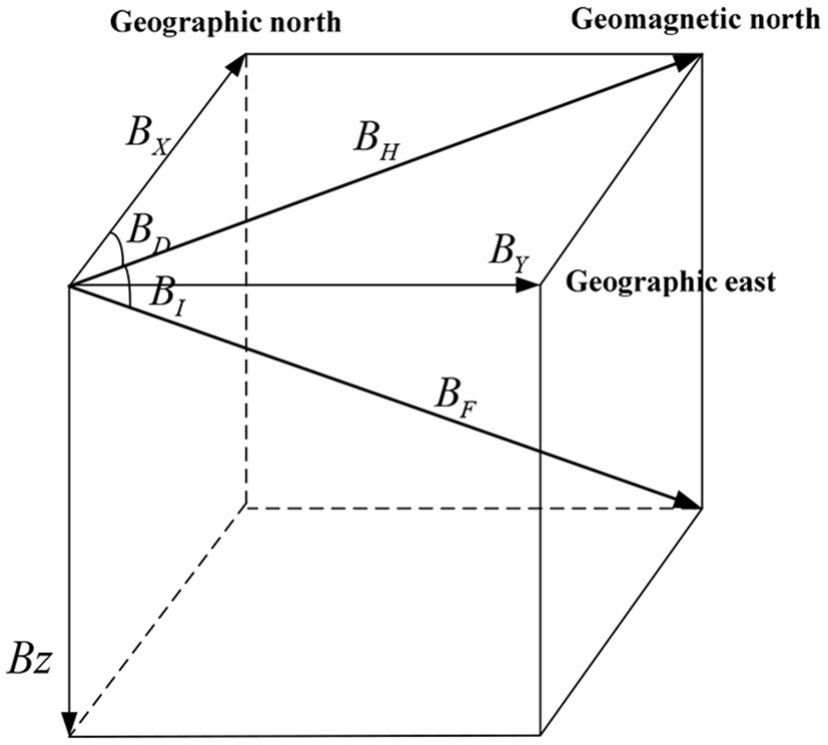
The vectors of the geomagnetic fields.

Geomagnetic fields are a very important cue for navigation by these animals (Zhang et al., 2019). At any point on the Earth's surface, geomagnetic fields can be described as vectors in three-dimensional space (see Figure 1). It is fairly well-known that the fields are derived from sources in the core and crust of the Earth, and the total geomagnetic field vectors have seven components, which can be resolved into the north component ***B***_***X***_, the east component ***B***_***Y***_, the vertical component ***B***_***Z***_, the horizontal component ***B***_***H***_, the total intensity component ***B***_***F***_, the declination angle component ***B***_***D***_, and the inclination angle component ***B***_***I***_. Thus, geomagnetic fields can provide very stable information about a location which animals can use to navigate.

Geomagnetic navigation technology can provide a reliable navigation method by measuring geomagnetic fields for mobile robots, such as underwater vehicle navigation (AUV) (Kinsey et al., 2006) and unmanned aircraft (UA) (Yuan et al., 2011; Kebria et al., 2020). Conventional geomagnetic navigation methods mainly focus on geomagnetic matching algorithms, which mainly include the Mean Absolute Difference algorithm (MAD) (Caifa et al., 2011), Mean Square Difference algorithm (MSD) (Rong, 2016), and Iterative Closest Contour Point algorithm (ICCP) (Lin et al., 2007; Xiao et al., 2019; Luo et al., 2021a). However, the conventional matching methods mainly depend on a priori geomagnetic map. There is a problem with the mentioned methods that the geomagnetic map needs to be drawn in advance (Ge and Zhou, 2007). To avoid any dependency on a priori geomagnetic map, the geomagnetic perceiving navigation method is proposed.

To satisfy the practical demand of geomagnetic navigation, the selection methods of characteristic components for geomagnetic matching were proposed based on statistical modeling (Qiao et al., 2007; Wei et al., 2010; Shang et al., 2019). The correlation research on the geomagnetic field parameters is indispensable for geomagnetic perceiving navigation, which introduced a new selection method for geomagnetic field parameters. We know that getting the right geomagnetic parameters affects the navigation result, and it is still important for navigational efficiency. This paper mainly discusses the correlation of geomagnetic field parameters for geomagnetic perceiving navigation technology.

In this paper, the similarity calculation based on the geomagnetic characteristics is a data association technology. For the correlation analysis between the geomagnetic parameters, the similarity calculation of the correlation coefficient is firstly introduced for geomagnetic perceiving navigation technology. Therefore, the correlation coefficient is calculated between the geomagnetic parameters, at the same time, the search algorithm (Hex-path algorithm) is used to verify the correlation analysis results. Simulation results of three cases are analyzed, and we can conclude that the same convergent state for the approximate correlation coefficient is apparent. In other words, the simulation results are in agreement with the similarity calculation results. In the future, we will focus on the research of geomagnetic perceiving navigation methods, and the present study is the preliminary preparation and the theoretical foundation for the follow-up work.

The rest of this paper is structured as follows: in section Correlation analysis on geomagnetic field parameters, the correlation analysis on the geomagnetic field parameters is introduced, and the results are given. In section Problem formulation of geomagnetic perceiving navigation, the search problem of geomagnetic perceiving navigation is raised. Next, the Hex-path algorithm is adopted in section Algorithm verification. Then, the simulation is introduced in section Results. Finally, the conclusion is given in section Conclusion.

## Correlation Analysis on Geomagnetic Field Parameters

Geomagnetic fields can be divided into two categories: geomagnetic intensity and geomagnetic angle. The geomagnetic intensity is mainly composed of the total geomagnetic intensity (***B***_***F***_), the horizontal intensity **(*B***_***H***_**)**, the north component (***B***_***X***_**)**, the east component **(*B***_***Y***_**)**, and the vertical component **(*B***_***Z***_**)**. The geomagnetic field angle is mainly composed of the geomagnetic declination angle ***B***_***D***_ and the geomagnetic inclination angle **(*B***_***I***_**)** (Zhao et al., 2014). Choosing suitable geomagnetic field parameters is the key to realizing geomagnetic perceiving navigation. Therefore, the selected geomagnetic parameters must have distinct statistical characteristics in the navigation area with high recognition and rich feature information.

**Figure 2:**
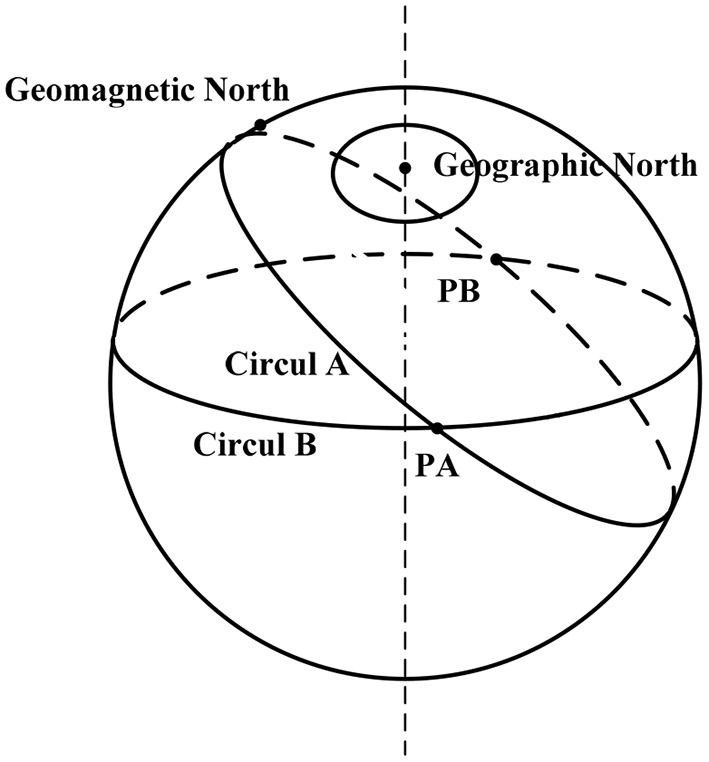
The uniqueness of geomagnetic fields.

If the Earth's geomagnetic field is an ideal magnetic dipole field, there will be two intersections PA and PB (see Figure 2) between the two geomagnetic contours in the whole Earth range, indicating that the geomagnetic components on these two points are the same (Jiang and Ran, 2011). It can be seen that only two geomagnetic characteristics fail to determine a unique geographical location on the Earth surface. Therefore, several geomagnetic characteristics are needed as geomagnetic parameters to ensure the unique position of the Earth surface.

The geomagnetic fields have seven parameters. There is a correlation between geomagnetic field parameters. In order to uniquely determine a position, we need to consider the independence of the three parameters from the seven vectors of the geomagnetic fields. Therefore, it is necessary to research the similarity degree of the seven vectors.

In essence, the similarity calculation based on the geomagnetic characteristics is a data association technology. The similarity between the geomagnetic characteristics mainly reflects the correlation of their variation characteristics. Therefore, the correlation coefficient is calculated by the similarity between multiple geomagnetic parameters, which are needed to take into account the global variation characteristics of the geomagnetic fields.

The data similarity calculation methods, which are commonly used in data association technology, mainly include the cosine similarity, the modified cosine similarity, and the correlation coefficient similarity (Saito et al., 1999; Luo et al., 2021b).

The three data similarity methods are followed as:

(i) The cosine similarity method can be described as:


(1) $$r_{ij}\;=\;cos\left(R_{i},R_{j}\right)=\frac{\sum_{i\neq j,i,j=1}^{n}r_{i}r_{j}}{\sqrt{\left[\sum_{i=1}^{n}r_{i}^{2}\right]\left[\sum_{j=1}^{n}r_{j}^{2}\right]}}$$

where ***R***_***i***_ and ***R***_***j***_ are the vector-evaluated parameters, respectively.

(ii) The modified cosine similarity method can be described as:


(2) $$r_{ij}\;=\;Adjust\;cos\left(R_{i},R_{j}\right)=\frac{\sum_{i\neq j,i,j=1}^{n}\left(r_{i}-\bar{r}\right)\left(r_{j}-\bar{r}\right)}{\sqrt{\left[\sum_{i=1}^{n}{\left(r_{i}-\bar{r}\right)}^{2}\right]}\sqrt{\left[\sum_{j=1}^{n}{\left(r_{j}-\bar{r}\right)}^{2}\right]}}$$

where $\bar{r}$ is the evaluation value of the vector-evaluated parameters.

(iii) The correlation coefficient similarity method can be described as:


(3) $$r_{ij}\;=\;Adjust\;cos\left(R_{i},R_{j}\right)=\frac{\sum_{i\neq j,i,j=1}^{n}\left(r_{i}-\bar{r_{i}}\right)\left(r_{j}-\bar{r_{j}}\right)}{\sqrt{\left[\sum_{i=1}^{n}{\left(r_{i}-\bar{r_{i}}\right)}^{2}\right]}\sqrt{\left[\sum_{j=1}^{n}{\left(r_{j}-\bar{r_{j}}\right)}^{2}\right]}}$$

where $\bar{r_{i}}$ and $\bar{r_{j}}$ are the evaluation values of the vector-evaluated parameters ***r***_***i***_ and ***r***_***j***_, respectively.

For the correlation analysis between the geomagnetic characteristics, the similarity calculation method of the correlation coefficient is firstly adopted. This paper proposes a method to calculate the similarity between several parameters by Euclidean distance. Therefore, the similarity calculation between geomagnetic characteristics can be expressed as follows:


(4) $$r_{XY}\;=\;\frac{\sum_{i\neq j,i,j=1}^{n}\left(x_{i}-\bar{x}\right)\left(y_{j}-\bar{y}\right)}{\sqrt{\left[\sum_{i=1}^{n}{\left(x_{i}-\bar{x}\right)}^{2}\right]}\sqrt{\left[\sum_{j=1}^{n}{\left(y_{j}-\bar{y}\right)}^{2}\right]}}$$

where ***X* = (*x***_**1**_**,*x***_**2**_**,…,*x***_***n***_**)** and ***Y* = (*y***_**1**_**,*y***_**2**_**,…,*y***_***n***_**)** are two parameter sequences, $\bar{x}$ and $\bar{y}$ are the evaluation values of the two parameter sequences, and ***r***_***XY***_ is the correlation coefficient between two geomagnetic parameters, −1 ≤ ***r***_***XY***_ **≤* 1***.

According to the correlation coefficient calculation method of data association, the degree of correlation between parameters can be divided into the following categories (Kong et al., 2012; Hu et al., 2019, 2020; Wu et al., 2020; Luo et al., 2021c):

1. If ***r***_***XY***_ **<** 0.3, the two parameters are irrelevant;
2. If −0.3 ≤ ***r***_***XY***_ **<** 0.5, the two parameters have a low degree of linear correlation;
3. If −0.5 ≤ ***r***_***XY***_ **<** 0.8, the two parameters are indicating significant linear correlation;
4. If 0.8 ≤ ***r***_***XY***_, the two parameters generally have highly linear correlation.

According to the distribution characteristics of geomagnetic parameters, the geomagnetic parameters ***B***_***X***_, ***B***_***Y***_, and ***B***_***Z***_ are different and independent from each other, so the correlation coefficient between the three parameters is 0 in theory.

If the correlation coefficient of any two geomagnetic parameters among the seven geomagnetic parameters is much larger than the correlation coefficient of the above three ($r_{XY}\;\geq \;r_{B_{X}↔B_{Y}},r_{XY}\;\geq \;r_{B_{X}↔B_{Z}},r_{XY}\;\geq \;r_{B_{Y}↔B_{Z}}$), then the two parameters have high similarity, and these parameters with high correlation coefficients are usually grouped into a class.

**Figure 3:**
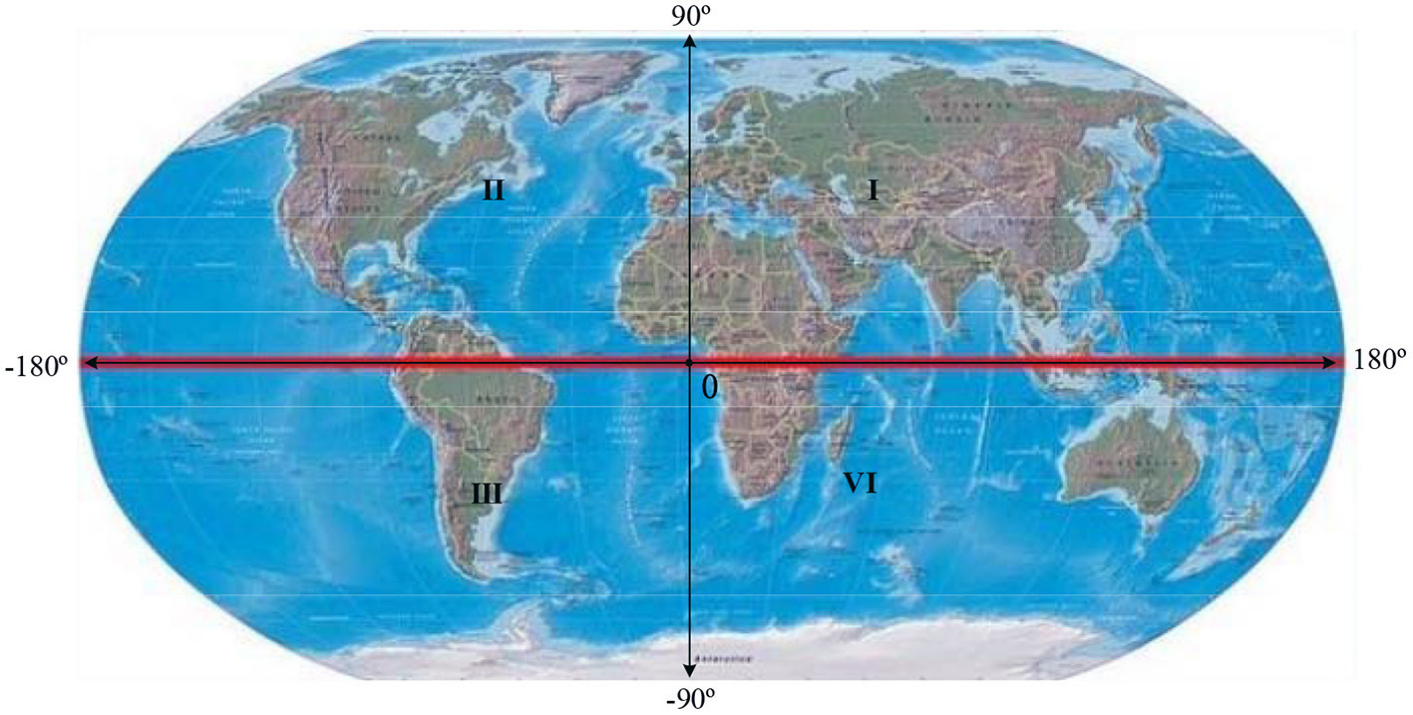
The map of the Earth's magnetic field.

The data analysis from the Word Magnetic Model (WMM2015) (Russell, 2004; Chulliat et al., 2015; Hu et al., 2021) pointed out the correlation of geomagnetic components on the Earth surface. For simplification of data statistics, the seven geomagnetic characteristics in the northern hemisphere of the Earth are taken as examples. The northern hemisphere is divided into the first quadrant I and the second quadrant II (see Figure 3).

The correlation coefficient of any two geomagnetic parameters can be calculated from the Word Magnetic Model database, and the results indicate that the seven parameters of the first and second quadrants of the northern hemisphere are divided into three groups as shown in Table 1.

**Table 1:** The correlation statistical results of geomagnetic parameters.

**Components**	**Correlation**	**Value**
**(*B*_*X*_ *B*_*H*_)**	***r*_*B*_*X*_↔*B*_*H*__**	0.9998
**(*B*_*Y*_ *B*_*D*_)**	***r*_*B*_*Y*_↔*B*_*D*__**	0.8365
**(*B*_*Z*_ *B*_*I*_ *B*_*F*_)**	***r*_*B*_*Z*_↔*B*_*I*__**&***r*_*B*_*Z*_↔*B*_*F*__**&***r*_*B*_*I*_↔*B*_*F*__**	0.9756 & 0.9666 & 0.8992

In geomagnetic perceiving navigation, the greater the correlation coefficient value between geomagnetic parameters, the higher the degree of the linear correlation between the two parameters, which can be equated to one category. As for the selection of the geomagnetic parameters, it is advisable to choose one with a small correlation coefficient.

According to the above analysis, the correlation of the geomagnetic parameters should be considered when selecting the geomagnetic parameters for navigation search. In other words, the geomagnetic components should be selected from the three groups, respectively, such as (***B***_***X***_***B***_***Y***_***B***_***Z***_), (***B***_***H***_***B***_***D***_***B***_***I***_), (***B***_***F***_***B***_***D***_***B***_***H***_), etc.

## Problem Formulation of Geomagnetic Perceiving Navigation

### Mathematical Model

Geomagnetic perceiving navigation is the search process of geomagnetic multi-parameters without a priori geomagnetic map. It indicates that an agent could only perceive the variation of the geomagnetic parameters to reach the destination by a geomagnetic sensor. The search behavior is the response to the geomagnetic environment stimuli, and its physical significance is that the geomagnetic multi-parameter could only determine geographic locations on the Earth (Liu et al., 2014). The process of geomagnetic perceiving navigation is the convergence process of the geomagnetic parameters from the start location to the target location. When the geomagnetic parameters converge to zero, it indicates that the agent has finished the navigation task. Therefore, the multi-objective convergence process can be considered as:


(5) $$\{\begin{array}{c} \min\;F\left(k\right)=(f_{1}\left(B,k\right),\;f_{2}\left(B,k\right),...\;,\;f_{n}\left(B,k\right))\\ s.t.:\;t_{i}\;=\;g_{i}\left(B,\theta^{k}\right)\leq \delta \end{array}$$

where ***k*** is the number of iterations, ***F***(**⦁**) is the objective function, ***f***(**⦁**) is the sub-objective function, **θ^*k*^** is the movement direction of an agent, ***g***(**⦁**) is the constraint condition, and **δ** is a preset value.

### The Normalization of the Objective Function

The search process of geomagnetic perceiving navigation presents a posteriori and temporal characteristics, and the convergence of the geomagnetic multi-parameters has a strong incentive and a restriction relationship with the motion behavior of an agent. Considering that the above process is a multi-objective posterior search, the objective function needs to be established for the optimization. Based on the characteristics of geomagnetic fields, the sub-objective function of the geomagnetic parameter search is constructed as:


(6) $$f_{i}\left(B,k\right)={\left[B_{i}\left(t\right)-B_{i}\left(k\right)\right]}^{2},\;1\leq i\leq 7$$

where ***B***_***i***_**(*t*)** is the ***i***th geomagnetic parameter of the target location and ***B***_***i***_**(*k*)** is the ***i***th geomagnetic parameter of the current location. There are different magnitudes and units within the geomagnetic field parameters, the objective function should be normalized as:


(7) $$F\left(k\right)=\frac{1}{N}\sum_{i=1}^{7}\frac{f_{i}\left(B,k\right)}{f_{i}\left(B,0\right)}\;=\;\frac{1}{N}\sum_{i=1}^{7}\frac{{\left[B_{i}\left(t\right)-B_{i}\left(k\right)\right]}^{2}}{{\left[B_{i}\left(t\right)-B_{i}\left(0\right)\right]}^{2}}$$

The purpose of the geomagnetic perceiving navigation is that the objective function could converge to the optimal value in the search process, which can be expressed as:


(8) ‖F(k)−F(k−1)‖≤ε

where **ε** is a preset value.

Based on the above description, the geomagnetic perceiving navigation problem could be generalized as the multi-objective posterior search problem, by calculating the objective function to find the optimal solution.

## Algorithm Verification

To analyze the correlation of the geomagnetic data, the Hex-path algorithm (Russell, 2004) will be adopted in the searching process of geomagnetic perceiving navigation. We know that the Hex-path algorithm has been used before, but only in odor source searching. The Hex-path odor searching algorithm could guide a mobile robot with a single gas sensor to search for an underground odor source. Here, the Hex-path algorithm is used to guide toward the target of lower objective function. Although the Hex-path algorithm is applied in different fields, the essence is the same, which means are all based on a change in the objective function. The major difference between the two is the fact that the Hex-path odor searching algorithm is guided toward regions of higher objective function. The reason for using this algorithm is the fact that it is simple and easy to implement step-by-step.

The Hex-path algorithm is implemented in the following steps:

**Step 1:** *Heading initialization*. Randomly generate a number of ***N*** initial individuals in the heading space ***Q***, which can be defined as ***Q*** **=** {**θ*_i_*|*i* = 1, 2,⋯, *N*** }. **θ*_i_*** can be expressed as:


(9) $$\theta_{i}\;=\;\Delta \theta \times j,j\in \left[1,m\right]$$

where $m\;=\;\frac{2\pi }{\theta }$ and **△***θ* is the sampling interval.

**Step 2:** *Heading selection*. Randomly select the sample θ*_i_*, and the probability of each selection is given by:


(10) $$p(\theta_{i})=\;\frac{1}{N}$$

**Figure 4:**
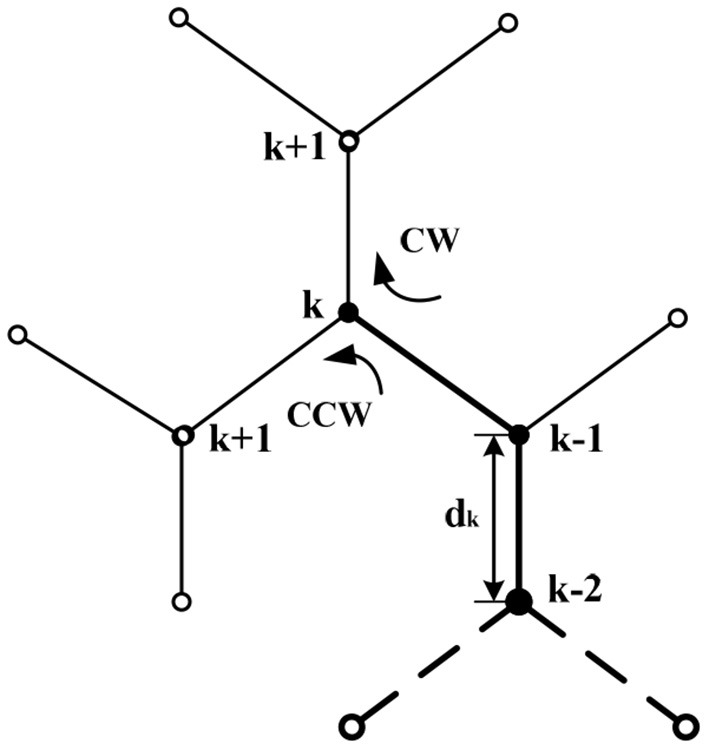
The turning selection diagram.

**Step 3:** *Heading updating rule*. The objective function has been calculated at points (***k*−2**) and (***k*−1**). If the objective function at (***k*−1**) decreases, it means that the target position is close to the right, and turning △*θ* to the clockwise (CW) direction could be performed. Otherwise, turning △*θ* to the counterclockwise (CCW) direction could be performed. The heading updating rule is shown in Figure 4.


(11) $$\{\begin{array}{c} \theta^{k+1}\;=\;\theta^{k}-\Delta \theta ,if\;F\left(k-1\right)\leq F\left(k-2\right)\;and\;d\text{θ}<0\\ \theta^{k+1}\;=\;\theta^{k}+\Delta \theta ,if\;F\left(k-1\right)>F\left(k-2\right)\;and\;d\text{θ}>0 \end{array}$$

where ***d***θ = θ***^k^***−θ***^k−1^***.

**Step 4:** *Terminate condition*. If the search algorithm meets the termination condition (12), the search algorithm will terminate; otherwise go to step (3) above.


(12) $$\{\begin{array}{c} \left\|F(k)-F(k-1)\right\|\leq \varepsilon \\ 0\;\leq \;g_{i}\left(B,\theta^{k}\right)\leq \delta \end{array}$$

## Results

To show the correlation of geomagnetic field characteristics, numerical simulations are implemented.

### Simulation Setup

The Word Magnetic Model (WMM2015) is used to provide real-time geomagnetic data. Simulations have been carried out based on the seven physical fields.

**Figure 5:**
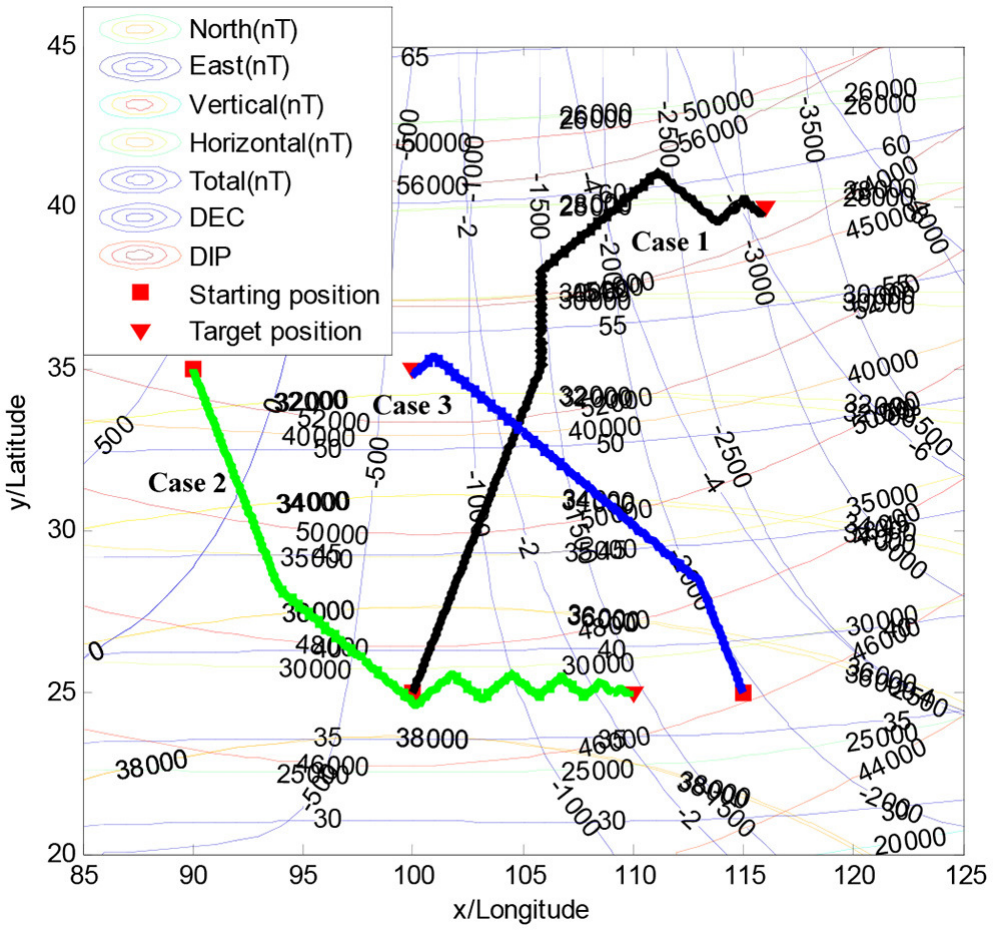
Geomagnetic perceiving navigation within the Hex-path algorithm.

In simulations, we choose a rectangular area from 20° north latitude and 85° west longitude (20N, 85W) to 45° north latitude and 125° west longitude (45N, 125W). In this scenario, the starting position is the red square “□,” and the target position is the red triangle “▽,” which are depicted in Figure 5. The simulation parameter are listed in Table 2.

**Table 2:** Setting navigation parameters.

**No**	**Parameters**	**Size**
1	△θ	60°
2	ε	0.001
3	δ	0.05
4	*d*_*k*_	10 km

### Simulation Results

To demonstrate the effectiveness of the Hex-path algorithm, several simulation results are given in this paper. As presented in Figure 5, three different starting positions and target positions are randomly selected.

Figure 5 illustrates the searching trajectory of the Hex-path algorithm, where the heading is updated continuously depending on the objective function at ***k*** and ***k*−1**. The searching results of the Hex-path algorithm present a good global search capability.

**Figure 6:**
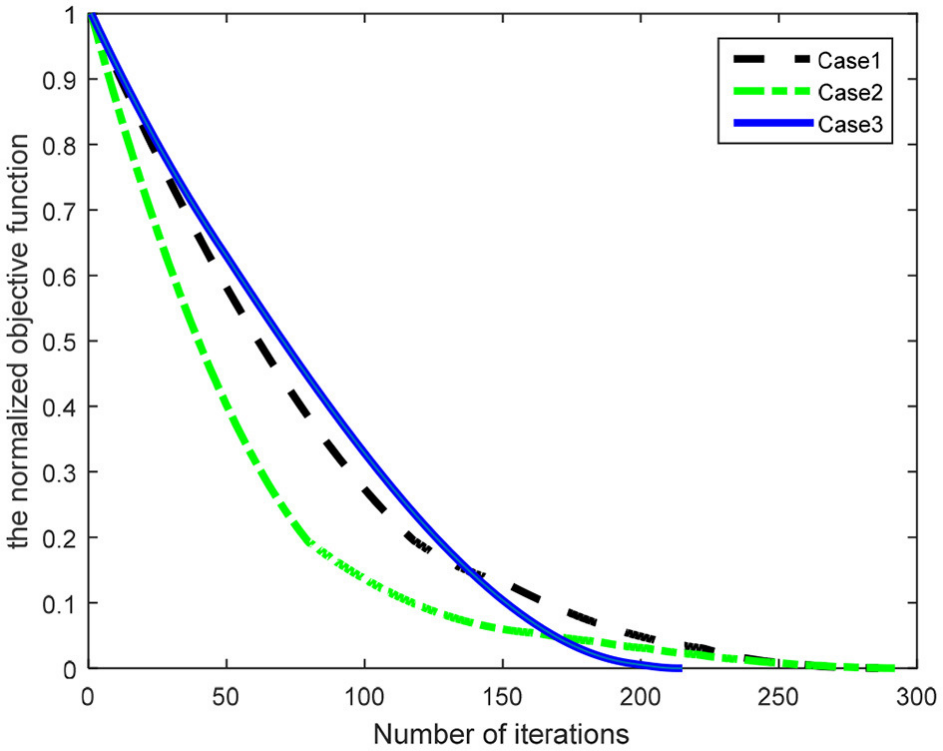
The convergence curves of the normalized objective function.

Figure 6 illustrates the convergence property of the normalized objective function in three cases, from which we can see that the number of iterations is 286, 292, and 215 for the Hex-path algorithm, respectively. The results show that the Hex-path algorithm could converge to a stable state.

The similarity in the number patterns of the normalized objective function shows the robustness of the Hex-path algorithm mentioned above. Figure 6 illustrates the steady states of the seven geomagnetic parameters.

### Analysis of the Results

**Figure 7:**
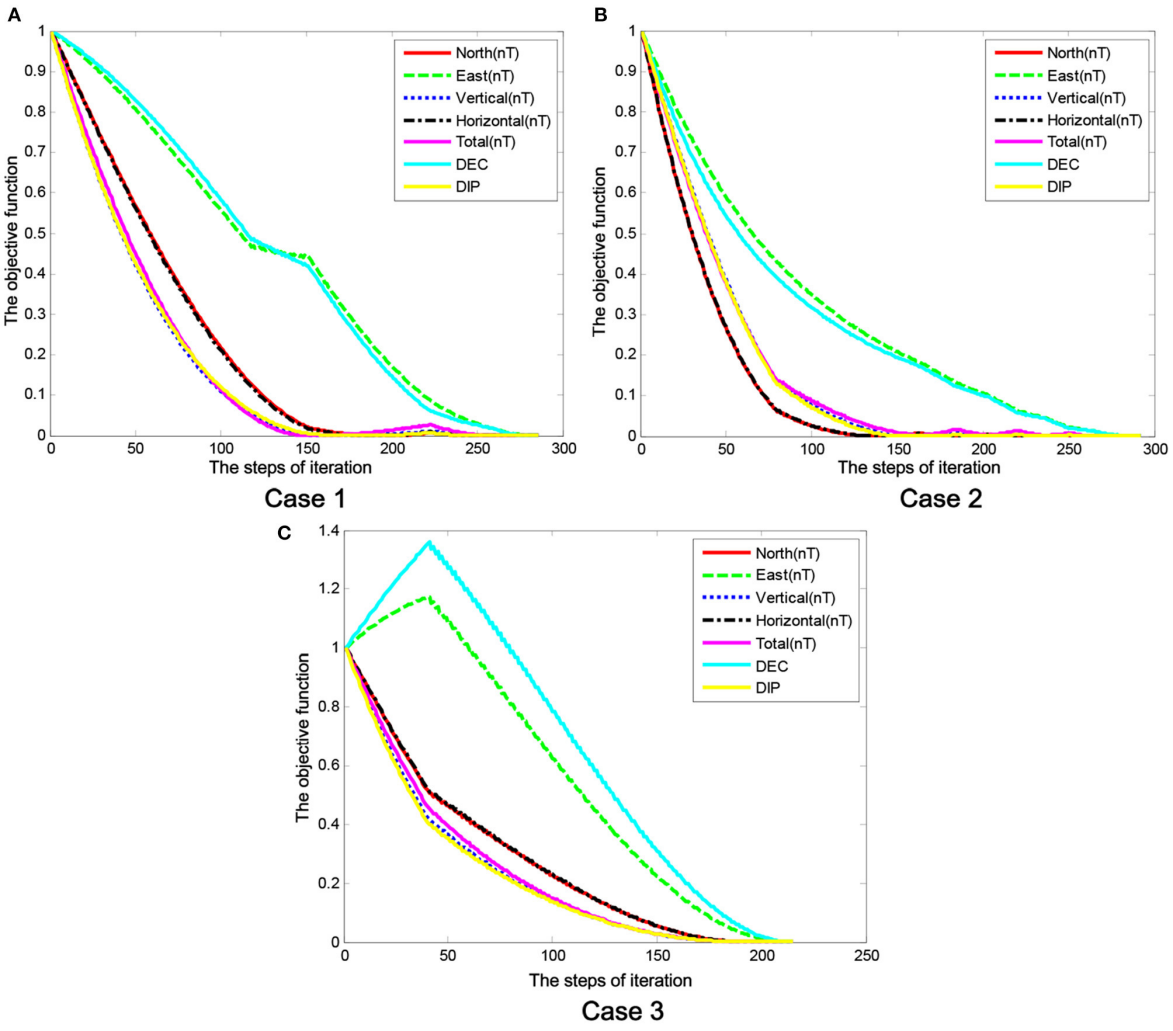
The geomagnetic multi-parameter convergence curves. **(A)** Case 1, **(B)** Case 2, and **(C)** Case 3.

Figure 7 shows the convergence property of the geomagnetic multi-parameter, wherein Figure 7A represents the above Case 1, Figure 7B represents the above Case 2, and Figure 7C represents the above Case 3. The convergence curves of the sub-objective function show a significant difference between the multi-parameters.

As can be seen in Figure 7, the variation rules of the geomagnetic multi-parameters reflect the correlation on the geomagnetic field parameters, where the north geomagnetic field component ***B***_***X***_ and the horizontal magnetic field component ***B***_***H***_ show the same convergent state of change, the east geomagnetic field component ***B***_***Y***_ and the declination angle ***B***_***D***_ show the same convergent state of change, and the vertical geomagnetic field component ***B***_***Z***_, the inclination angle ***B***_***I***_, and the total intensity ***B***_***F***_ show the same convergent state of change. Meanwhile, the same results can be seen in Table 1.

From the above analyses, we clearly see that the Hex-path algorithm ensures the desired target position depends on geomagnetic environment stimuli during the training iteration, and the learning task of each iteration also depends on those of the completed ones. The similarity in the number patterns of the objective function shows the consistency of the convergence trend on the correlation analysis results mentioned above.

## Conclusion

This paper discusses the correlation of geomagnetic field parameters, and the simulations show the same results as in Table 1. The results show the same convergent state for the approximate correlation coefficient. In other words, the approximate correlation coefficient between the geomagnetic parameters can be regarded as one class. The correlation study on geomagnetic field parameters is crucially important for geomagnetic navigation technology and introducing the selection and application of geomagnetic field parameters.

In the future, we will focus on the research of geomagnetic perceiving navigation methods, but the study of the correlation of geomagnetic field parameters is very necessary for geomagnetic perceiving navigation technology. We know that getting the right geomagnetic parameters might affect the navigation result, and it is still important for navigational efficiency. Therefore, this paper mainly discusses the correlation of geomagnetic field parameters for geomagnetic perceiving navigation technology. The present study is the preliminary preparation and the theoretical foundation for the follow-up work.

## Data Availability Statement

The original contributions presented in the study are included in the article/supplementary material, further inquiries can be directed to the corresponding author/s.

## Author Contributions

HL, JQ, XJ, and YN designed and established the theoretical model. HL wrote the paper and performed the experiments. JQ provided some ideas to improve and perfect the paper. YN analyzed the data. XJ reviewed and edited the manuscript. All authors read and approved the manuscript.

## Funding

This work was supported by the Scientific Research Project of Education Department of Shaanxi Province Fund under grant 20JK0915, the General Special Project of Shaanxi Science and Technology Department under Grant No. 2021JQ-714, the XI'AN Key Laboratory of Advanced Control and Intelligent Process under Grant No. 2019220714SYS022CG044, and the Laboratory of Science and Technology on Marine Navigation and Control under Grant No. 2021010106.

## Conflict of Interest

The authors declare that the research was conducted in the absence of any commercial or financial relationships that could be construed as a potential conflict of interest.

## Publisher's Note

All claims expressed in this article are solely those of the authors and do not necessarily represent those of their affiliated organizations, or those of the publisher, the editors and the reviewers. Any product that may be evaluated in this article, or claim that may be made by its manufacturer, is not guaranteed or endorsed by the publisher.

## References

[B1] Caifa G. Li A. Hong C. Yang H. (2011). “Algorithm for geomagnetic navigation and its validity evaluation,” in International Conference on Computer Science and Automation Engineering (CSAE), Shanghai, 573–577.

[B2] Chulliat A. Macmillan S. Alken P. Beggan C. Nair M. Hamilton B. (2015). The US/UK World Magnetic Model for 2015-2020. Available online at: http:no-ra.nerc.ac.uk/18737/

[B3] Dennis T. E. Rayner M. J. Walker M. M. (2007). Evidence that pigeons orient to geomagnetic intensity during homing. Proc. R. Soc. B Biol. Sci. 274, 1153–1158. 10.1098/rspb.2007.3768 17301015 PMC2189574

[B4] Ge Z. Zhou J. (2007). “A new approach to geomagnetic matching navigation,” in International Conference on Spatial Information Technology, Wuhan, 67952–67956. 10.1117/12.77482917645476

[B5] Hu L. Chan K. Yuan X. Xiong S. (2019). “A variational bayesian framework for cluster analysis in a complex network,” in IEEE Transactions on Knowledge and Data Engineering. 10.1109/TKDE.2019.291420027295638

[B6] Hu L. Pan X. Tan Z. Luo X. (2021). A fast fuzzy clustering algorithm for complex networks via a generalized momentum method. IEEE Trans. Fuzzy Syst. 14, 1–13. 10.1109/TFUZZ.2021.311744227295638

[B7] Hu L. Zhang J. Pan X. Yan H. You Z. (2020). HiSCF: leveraging higher-order structures for clustering analysis in biological networks. Bioinformatics 37, 542–550. 10.1093/bioinformatics/btaa775 32931549

[B8] Jiang L. Ran L. (2011). “Pure geomagnetic homing navigation on earth surface,” in International Conference on Electronics, Communications and Control (ICECC), Ningbo, 971–974. 10.1109/ICECC.2011.606672027295638

[B9] Kebria P. M. Khosravi A. Salaken S. M. Nahavandi S. (2020). Deep imitation learning for autonomous vehicles based on convolutional neural networks. IEEE/CAA J. Autom. Sinica 1, 82–95. 10.1109/JAS.2019.191182527295638

[B10] Kinsey J. C. Eustice R. M. Whitcomb L. L. (2006). A survey of underwater vehicle navigation: Recent advances and new challenges. IFAC Conf. Manoeuver. Contr. Mar. Craft 88, 20090–20102. 10.1.1/134.5601

[B11] Kong L. Qin K. Long T. (2012). Global SST data mining based on fuzzy clustering. Geomat. Inform. Sci. Wuhan Univer. 37, 215–219. 10.13203/j.whugis2012.02.027

[B12] Kramer G.. (1953). Wird die Sonnenhhe bei der Heimfinderorientierung verwertet? J. Ornithol. 94, 201–219. 10.1007/BF01922508

[B13] Lin Y. Yan L. Tong Q. (2007). “Underwater geomagnetic navigation based on ICP algorithm,” in IEEE International Conference on Robotics and Biomimetics, Sanya, 2115–2120.

[B14] Liu M. Liu K. Peng X. Hong L. (2014). “Bio-inspired navigation based on geomagnetic for the autonomous underwater vehicle,” in Oceans 2014 – Taipei, Taipei, 1–5. 10.1109/OCEANS-TAIPEI.2014.696444628747884

[B15] Luo X. Liu Z. L. Jin Z. Y. Zhou M. (2021a). Symmetric nonnegative matrix factorization-based community detection models and their convergence analysis. IEEE Trans. Neural Netw. Learn. Syst. 10, 1–13. 10.1109/TNNLS.2020.3041360 33513110

[B16] Luo X. Qin W. Dong A. Sedraoui K. Zhou M. C. (2021b). Efficient and high-quality recommendations via momentum-incorporated parallel stochastic gradient descent-based learning. IEEE/CAA J. Automat. Sinica 8, 402–411. 10.1109/JAS.2020.100339627295638

[B17] Luo X. Zhou Y. Liu Z. Hu L. Zhou M. (2021c). Generalized nesterov's acceleration-incorporated non-negative and adaptive latent factor analysis. IEEE Trans. Serv. Comput. 99, 1–14. 10.1109/TSC.2021.306910827295638

[B18] Qiao Y. Wang S. Qi Z. (2007). Selection of the characteristic variable of geomagnetism for matching. Seismol. Geomagnet. Observ. Res. 28, 42–47. 10.3969/j.issn.1003-3246.2007.01.007

[B19] Rodda G. H.. (1984). The orientation and navigation of juvenile alligators: evidence of magnetic sensitivity. J. Comp. Physiol. 154, 649–658. 10.1007/BF01350218

[B20] Rong M. O.. (2016). “Studying on comparison of different geomagnetic matching navigation algorithms,” in Geomatics and Spatial Information Technology, 1–5.

[B21] Russell R. A.. (2004). “Chemical source location and the RoboMole project,” in Proceedings of the Australasian Conference on Robotics and Automation, Canberra.

[B22] Saito H. Jin C. Ishio S. (1999). Principle of magnetic field analysis by MFM signal transformation and its application to magnetic. IEEE Trans. Magnet. 35, 3992–3992. 10.1109/20.80073227295638

[B23] Shang M. S. Luo X. Liu Z. Chen J. Yuan Y. Zhou M. C. (2019). Randomized latent factor model for high-dimensional and sparse matrices from industrial applications. IEEE/CAA J. Automat. Sinica 6, 131–141. 10.1109/JAS.2018.751118927295638

[B24] Walker M. M. Dennis T. E. Kirschvink J. L. (2002). The magnetic sense and its use in long-distance navigation by animals. Curr. Opin. Neurobiol. 12, 735–744. 10.1016/S0959-4388(02)00389-6 12490267

[B25] Walker M. M. Diebel C. E. Haugh C. V. Pankhurst P. M. Montgomery J. C. Green C. R. (1997). Structure and function of the vertebrate magnetic sense. Nature 390, 371–376. 10.1038/37057 20358649

[B26] Wei Q. I. Wang X. F. Xi-Hai L. I. Liu D. Z. (2010). Selection of characteristic components for geomagnetic matching based on statistical modeling. Progr. Geophys. 25, 324–330. 10.1017/S000497271000177230886898

[B27] Wu H. Luo X. Zhou M. C. (2020). Advancing non-negative latent factorization of tensors with diversified regularizations. IEEE Trans. Serv. Comput. 8, 1–13. 10.1109/TSC.2020.298876027295638

[B28] Xiao J. Duan X. Qi X. Liu Y. (2019). An improved ICCP matching algorithm for use in an interference environment during geomagnetic navigation. J. Navigat. 73, 1–19. 10.1017/S037346331900053530886898

[B29] Yuan S. Zhang J. Wang S. Wei J. (2011). “Research on real-time route planning for unmanned aircraft in geomagnetic matching guidance,” in IEEE. International Conference on Mechatronics and Automation, Beijing, 197–202.

[B30] Zhang Y. Liu X. Luo M. Yang C. (2019). Bio-Inspired approach for long-range underwater navigation using model predictive control. IEEE transactions on cybernetics. 2019, 2933–2937. 10.1109/TCYB.2019.2933397 31449042

[B31] Zhao Z. Hu T. Cui W. Huangfu J. Li C. (2014). Long-Distance geomagnetic navigation: imitations of animal migration based on a new assumption. IEEE Trans. Geosci. Remote Sens. 52, 6715–6723. 10.1109/TGRS.2014.230144127295638

